# Utility of whole‐body diffusion‐weighted magnetic resonance imaging in the management of treatment‐related neuroendocrine prostate cancer

**DOI:** 10.1002/iju5.12242

**Published:** 2020-11-29

**Authors:** Ryo Kurashina, Toshiki Kijima, Akihito Okazaki, Hirotaka Fuchizawa, Issei Suzuki, Kazumasa Sakamoto, Hironori Betsunoh, Yoshitatsu Fukabori, Masahiro Yashi, Takao Kamai

**Affiliations:** ^1^ Department of Urology Dokkyo Medical University Tochigi Japan

**Keywords:** chemotherapy, diffusion‐weighted magnetic resonance imaging, neuroendocrine tumors, prostate cancer, prostate‐specific antigen

## Abstract

**Introduction:**

Treatment‐related neuroendocrine prostate cancer, a rare and aggressive malignancy that emerges during androgen deprivation therapy characterized by low serum prostate‐specific antigen concentrations, is challenging to monitor because it is associated with predominantly visceral and lytic bone metastases.

**Case presentation:**

We describe the case of a 69‐year‐old man with treatment‐related neuroendocrine prostate cancer in whom the treatment response could be monitored using whole‐body diffusion‐weighted magnetic resonance imaging in addition to serum concentrations of neuroendocrine markers. The patient responded well to platinum‐based chemotherapy and achieved a complete response, as evidenced by these diagnostic modalities.

**Conclusion:**

Our case suggests that whole‐body diffusion‐weighted magnetic resonance imaging is useful in disease management for treatment‐related neuroendocrine prostate cancer as well as the potential evaluation of mixed responses and treatment resistance.

**Abbreviations & Acronyms**
ADC: apparent diffusion coefficient
ADT: androgen deprivation therapy
BSI: bone scan index
DWI: diffusion‐weighted magnetic resonance imaging
EP: etoposide‐cisplatin
FDG: fluorodeoxyglucose
MRI: magnetic resonance imaging
NSE: neuron‐specific enolase
PET: positron emission tomography
ProGRP: pro‐gastrin‐releasing peptide
PSA: prostate‐specific antigen
PSMA: prostate‐specific membrane antigen
t‐NEPC: treatment‐related neuroendocrine prostate cancer
WB: whole‐body

**Keynote message**

t‐NEPC, a rare and aggressive form of prostate cancer, may arise after ADT. Our case findings suggest that t‐NEPC may be well controlled with platinum‐based chemotherapy if its development is identified early and in a timely manner. Longitudinal monitoring of serum neuroendocrine marker concentrations and WB‐DWI findings would enable the response evaluation of t‐NEPC.

## Introduction

Neuroendocrine prostate cancer is a rare and aggressive malignancy associated with a low PSA concentration and poor response to ADT.^1^ Although it may arise de novo, most cases arise via neuroendocrine differentiation in response to ADT.^2^ The development of highly potent androgen receptor‐targeted agents has increasingly emphasized the importance of t‐NEPC.^3^, ^4^ However, treatment response monitoring in patients with t‐NEPC is sometimes difficult because the disease is associated with predominantly visceral and lytic bone metastases that cannot be evaluated using bone scintigraphy; moreover, disease activity does not correlate with serum PSA levels.

DWI, a novel imaging technique that provides both quantitative (e.g. ADC) and qualitative (e.g. signal intensity) data, can be used to distinguish malignant from benign lesions. Recently, WB‐DWI has emerged as a new technique for assessing the systemic spread^5^ and the treatment responses of diseases such as prostate cancer.^6^, ^7^

Here, we describe a case of t‐NEPC in which treatment response was monitored using WB‐DWI in addition to serial serum neuroendocrine marker monitoring.

## Case presentation

**Fig. 1:**
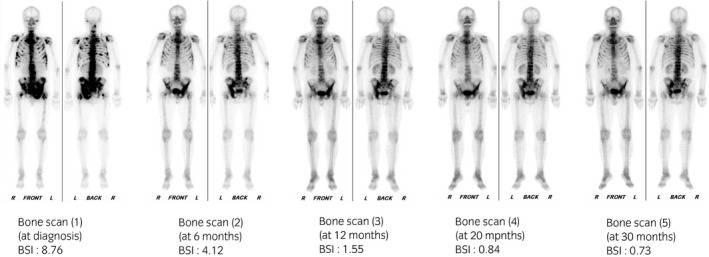
Bone scan findings and BSI during ADT.

A 69‐year‐old man with a history of hypertension previously visited another hospital complaining of lumbar pain. He was referred to our hospital for further examination. At our institution, his serum PSA concentration had increased to 163 ng/mL (normal range: 0–4 ng/mL); a digital rectal examination revealed a stony hard nodule suggestive of prostate cancer. A CT scan revealed multiple sclerotic bone metastases and metastases at the intrapelvic lymph nodes. Bone scintigraphy also identified multiple bone metastases at the vertebrae, ribs, and pelvic bone (extent of disease^8^ 3). A prostate biopsy revealed a Gleason score of 5 + 4 adenocarcinoma, leading to a diagnosis of prostate cancer, clinical stage T3bN1M1b. The patient received ADT with gonadotropin‐releasing hormone antagonist (degarelix) because first‐line docetaxel or abiraterone acetate was not approved in Japan at that time. After the initial ADT, his PSA values declined to undetectable levels (<0.008 ng/mL) and his symptoms disappeared. Bone scintigraphy revealed improvements in the bone lesions with a decreasing trend in the BSI (Fig. 1).

**Fig. 2:**
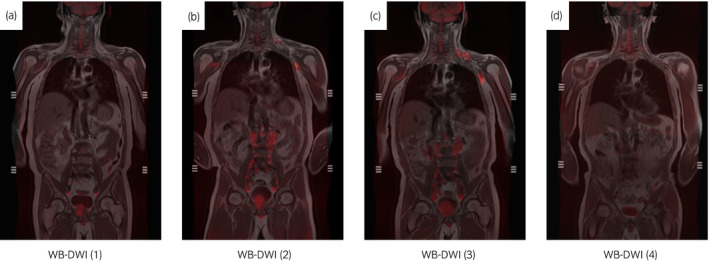
WB‐DWI findings before and after EP chemotherapy. Images are fusion images of DWI signals (shown in red) and morphological images (T1‐weighted images).

Although he was free from lumbar pain and his serum PSA concentration remained at undetectable levels, he noticed difficulty in urination 30 months after ADT. Bone scintigraphy revealed a stable disease with a BSI of 0.73, and there was no change in sclerotic appearance of bone lesions. As development of t‐NEPC at the primary lesion was suspected, WB‐DWI was performed with the measurement of serum neuroendocrine markers NSE and ProGRP. WB‐DWI revealed an enlarged prostate with a high signal intensity, suggestive of progressive disease at the primary lesion (Fig. 2a); however, the NSE and ProGRP levels were within normal range (<16.3 ng/mL and <81.0 pg/mL, respectively).

**Fig. 3:**
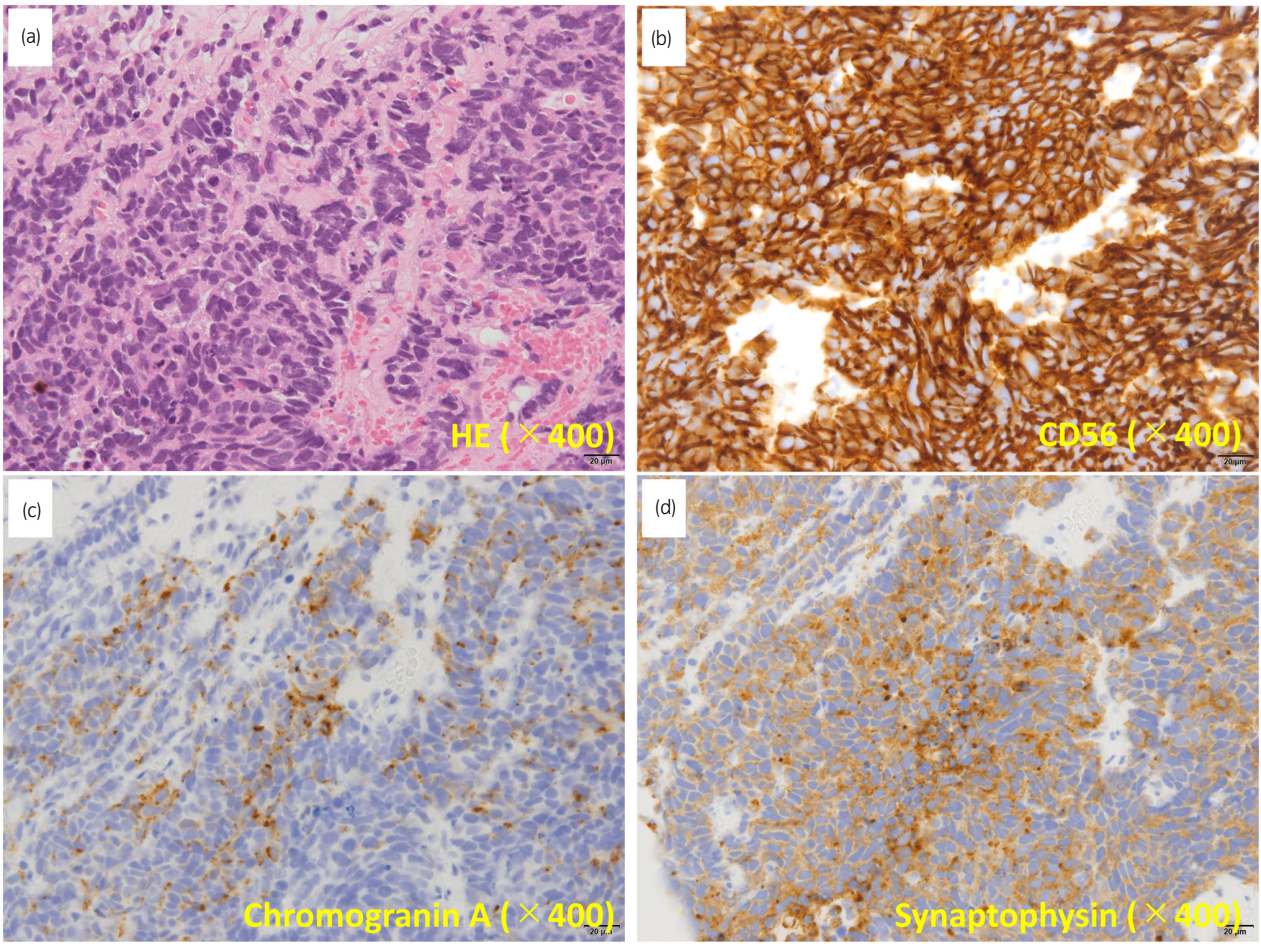
Tissue analyses of prostate biopsy specimens. (a) Hematoxylin and eosin staining and immunohistochemistry for (b) CD56, (c) chromogranin A, and (d) synaptophysin.

The patient remained on ADT; however, his serum neuroendocrine markers began to increase at 31 months. WB‐DWI at 36 months revealed progression of the primary site and newly developed pelvic, abdominal, and mediastinal lymph node metastases, although the bone lesions had no abnormal signal intensity on WB‐DWI (Fig. 2b) and still showed sclerotic appearance on CT (Fig. S1). A prostate rebiopsy was performed; histological analysis revealed small cell carcinoma. Immunohistochemistry analysis revealed positive staining for the neuroendocrine markers CD56, synaptophysin, and chromogranin A (Fig. 3).

**Fig. 4:**
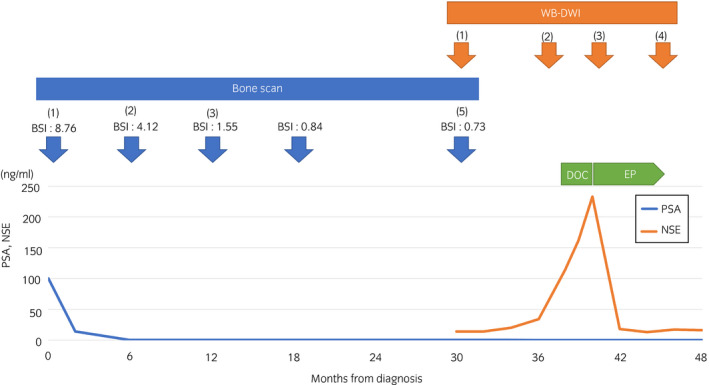
Treatment course and trends in the serum concentrations of PSA and NSE.

Docetaxel therapy was initially introduced; however, NSE (233.4 ng/mL) and ProGRP (128 pg/mL) further increased after one cycle of docetaxel and WB‐DWI showed no improvement in lymph node metastases (Fig. 2c). The patient received EP therapy comprising etoposide (100 mg/m^2^) on days 1–3 and cisplatin (80 mg/m^2^) on day 1, every 21 days. After four cycles of EP therapy, the patient’s serum neuroendocrine markers normalized and the abnormal signals on WB‐DWI disappeared completely (Figs 2 and 4), indicating a complete response. EP therapy was interrupted accordingly. The patient continued to receive ADT and denosumab and remained recurrence‐free 10 months after the EP therapy was interrupted.

## Discussion

In this report, we described the usefulness of WB‐DWI for treatment response evaluation of t‐NEPC. This malignancy has a poor prognosis; a literature review reported a median survival period of only 7 months after diagnosis.^9^ A platinum‐based chemotherapy regimen similar to that used to treat lung small cell carcinoma has been widely administered to patients with t‐NEPC. However, the most commonly used regimen (cisplatin or carboplatin combined with etoposide) yielded an objective response rate and median overall survival of only 8.9% and 9.6 months, respectively.^10^ In our case, although multiple sclerotic bone metastases responded well to initial ADT and remained sclerotic, WB‐DWI at the time of t‐NEPC detection revealed abnormal signal intensity only at the primary lesion and the lymph nodes. This finding suggested that t‐NEPC development was detected at a relatively early stage. Moreover, the disappearance of the abnormal signals on WB‐DWI enabled the safe interruption of chemotherapy, which allowed us to avoid adverse effects without compromising efficacy. Hence, we believe that WB‐DWI, which enabled an optimal response evaluation, contributed to a better outcome in our patient.

MRI, which does not expose patients to ionizing radiation,^11^ is particularly attractive for the repeated monitoring of cancer patients. Using contemporary MRI machines, WB‐DWI can be acquired with slightly longer time (<30 min), but without any additional cost, contrast medium, or special equipment. In addition to excellent soft tissue contrast, WB‐DWI^5^ can evaluate the systemic spread of malignant disease.^12^ Evidence suggests that the sensitivity of WB‐DWI for bone and soft tissue metastasis detection is comparable to that of FDG‐PET, and both are significantly more accurate than conventional CT and bone scans.^13^ WB‐DWI, which combines size, morphologic data, and ADC values, can be used to assess treatment responses.^14^

In our case, we decided to terminate docetaxel therapy after one cycle and changed to EP therapy that was shown to be very effective thereafter. This early treatment change was enabled by WB‐DWI that is not associated with radiation, contrast medium, and additional cost, and could be performed repeatedly for treatment response monitoring. This case illustrated the usefulness of WB‐DWI not only in early disease detection, but also in treatment response monitoring.

The utility of PET in clinical management in t‐NEPC has also been reported. Spratt *et al*. reported that FDG‐PET was useful in the detection of metastatic disease as well as treatment response evaluation in t‐NEPC.^15^ Recently, PET using tracer targeting PSMA has been developed, and PSMA‐PET provides higher detection rates than does FDG‐PET in prostate adenocarcinoma.^16^ For t‐NEPC, however, utility of PSMA‐PET is limited owing to neuroendocrine differentiation in which tumor cells express somatostatin receptors instead of PSMA.^17^ The potential complementary role of WB‐DWI and FDG‐PET in clinical management in t‐NEPC needs to be evaluated in future studies.

In conclusion, consistent with previous reports demonstrating the clinical usefulness of WB‐DWI for response evaluation in prostate cancer patients,^6^, ^7^ WB‐DWI was useful in management of t‐NEPC in this patient. WB‐DWI may also be useful in evaluation of mixed responses and treatment resistance.

## Conflict of interest

The authors declare no conflict of interest.

## Supporting information


**Figure S1**. CT scan finding of bone metastases at diagnosis and at detection of t‐NEPC.
