# Supplementary figures and images for: Utility of whole‐body diffusion‐weighted magnetic resonance imaging in the management of treatment‐related neuroendocrine prostate cancer
Source: IJU Case Rep. 2020 Nov 29;4(2):69–73. doi: 10.1002/iju5.12242 (PMC7924092; doi:10.1002/iju5.12242)

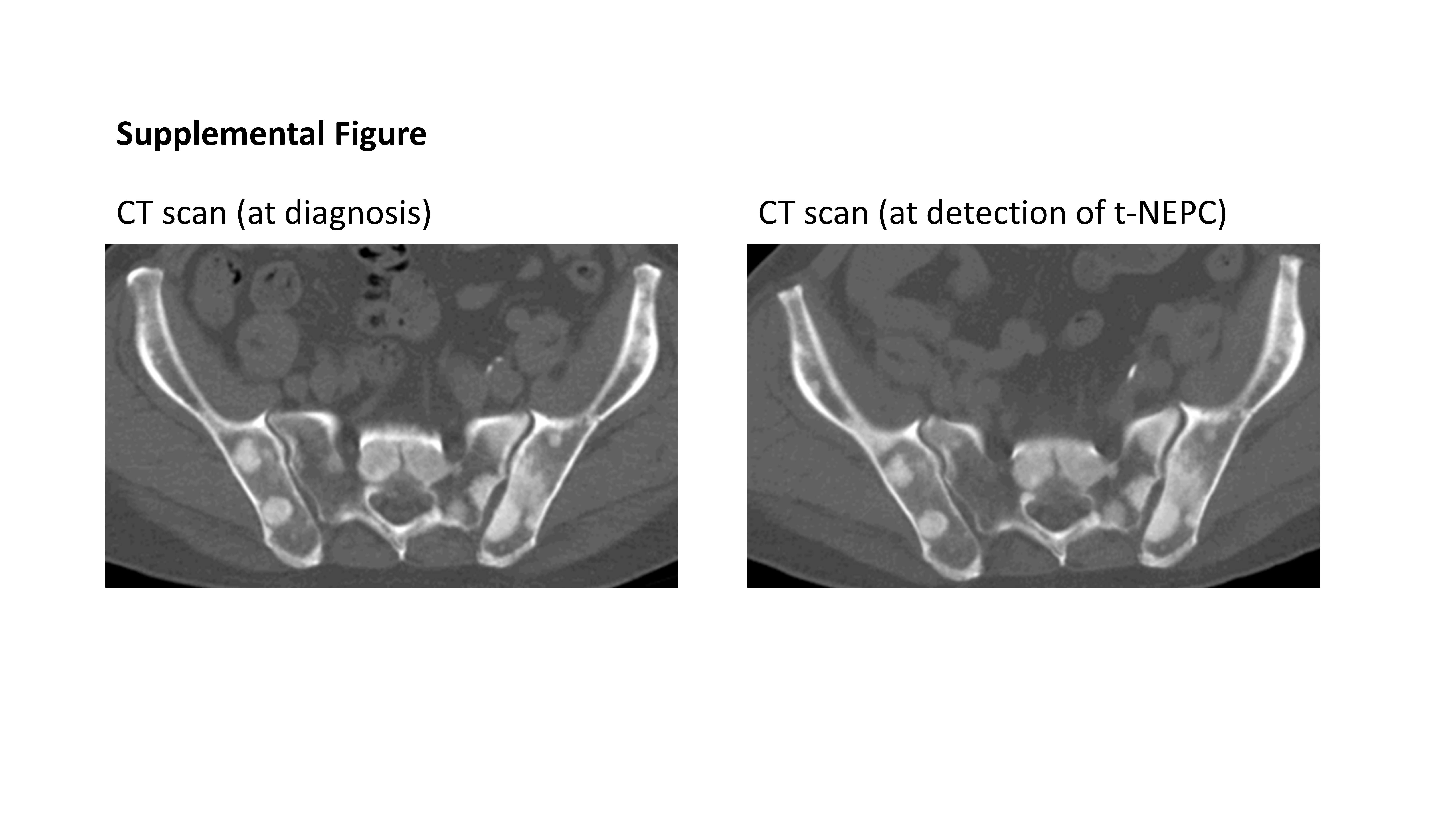

Supplement: Supplementary file 1 — Figure S1. CT scan finding of bone metastases at diagnosis and at detection of t‐NEPC. [file IJU5-4-69-s001.tif]
